# Dealing with Evidence in Dental Professional Liability Lawsuits—General Recommendations for Dental Expert Witness Work Using the Example of Germany: A Narrative Review

**DOI:** 10.1055/s-0044-1788320

**Published:** 2024-07-16

**Authors:** Hans Ulrich Brauer, Andreas Bartols

**Affiliations:** 1 Dental Academy for Continuing Professional Development, Karlsruhe, Germany; 2 Clinic for Conservative Dentistry and Periodontology, Christian-Albrechts-University Kiel, Kiel, Germany

**Keywords:** medical liability, standard of care, evidence, research, peer-reviewed dental, guidelines, expert opinion

## Abstract

Dental treatment can lead to disputes between patients and dentists, which are then brought before the civil courts as medical malpractice cases. The court regularly commissions a dental expert to answer questions of evidence in court. In the majority of cases, the expert is able to answer these questions based on his professional expertise and practical experience. In order to make the assessment comprehensible and credible for the judge and the parties, it can be helpful and reasonable to cite relevant literature references from dental textbooks and specialist journals. In individual cases, it may be necessary to carry out a systematic literature search on specific topics. Based on the situation in Germany, this narrative review makes recommendations of how to perform a literature search and make citations specifically for expert opinions that are generally applicable independent of national jurisdiction.

## Introduction

### Dental Liability Lawsuits

Dental prosthetic treatment does not always end to the satisfaction of the patient. 1 2 In fact, it can be the starting point for conflicts. Conflicts can of course also arise after a complication and/or when permanent damage to health has occurred. What these conflicts often have in common is that they start with a lack of trust or a loss of trust on the part of the patient in their dentist. 2 In many countries worldwide, these disputes can be brought before a civil court as medical malpractice claims. 2 3 In recent years, the number of legal disputes against dentists has increased worldwide. 4 5 6 7 The reasons for this increase in civil lawsuits resulting from dental treatment are complex. 3 5 8 One common reason is the patient's own financial contribution or the complete assumption of treatment costs. High costs are usually also coupled with high expectations, which can be triggered by advertising and the competitive situation of dentists. 3 In addition, the increasing number of lawyers specializing in medical law and a generally increased willingness on the part of patients to file a lawsuit against the doctor (possibly flanked by legal expenses insurance) can also play a role. 5 7 In dental liability lawsuits, the plaintiffs predominantly are the patients. A frequent reason in dental assessment practice is, for example, that a patient is dissatisfied with the technical or esthetic design of a newly incorporated prosthetic reconstruction or has difficulties with handling a newly fabricated denture. Cases of this kind are often not easy to objectify and are not immediately comprehensible to the expert. Much clearer are claims for damages in cases where objectifiable damage to health has occurred, such as after unilateral, permanent anesthesia following local anesthesia in the lip and/or tongue or the development of an abscess following oral surgery, which subsequently required hospitalization. 9 10 11 Another aspect that is frequently a matter of dispute in dental liability lawsuits is a violation of the duty to inform the patient. 12 Breaches of the duty to provide information are criticized particularly in the case of complications arising from implantological procedures, for example, following injury to the inferior alveolar nerve during implant placement in the lower jaw. 13 In a retrospective study of court opinions in maxillofacial surgery, it was found that in 33.1% of the cases evaluated, lack of information was the reason for the complaint. 14

However, all disciplines of dentistry are affected by civil lawsuits. 4 6 12 15 16 17 18 19 20 21 In the field of implantology, liability claims following altered sensation in the mandible after implant surgery dominate. 19 Similarly, bleeding during oral surgery and endodontic therapies in general are frequently cited as a cause of action. 20 21

If the treatment fee is not paid, the dentist is typically the party bringing the action. 16 This constellation is referred to as a fee claim. 22

### The Specific Context of Germany

The legal situation in Germany is comparable in many Western countries, despite some differences of the legal systems. This means that no special national route is chosen for medical malpractice cases in Germany as opposed to Sweden and New Zealand, for example, where the compensation for victims of medical malpractice is mainly handled by external committees and not by the courts. 23 If a legal dispute arises, the dentist's liability insurance and—if available—the patient's legal insurance often have an influence on the further legal dispute. 2 For the dentist involved, this means that the treatment case is shifted to a level outside of their profession. 2 In Germany, depending on the amount in dispute, the case is handled by the local district court or the competent regional court (amount in dispute higher than 5,000 Euros). In civil proceedings, one or more judges of a senate deal with the dispute. Since the judges do not have specialist dental knowledge, they usually define a topic of evidence with specific questions of evidence. The judges thus delegate the task of answering the questions posed to a qualified dental expert. 1 This is comparable to other countries. 6 24 In Germany, expert evidence is regulated in paragraphs 402 et seq. of the Code of Civil Procedure (ZPO). 25 It is also possible that social courts or, in even rarer cases, criminal courts may require a dental expert.

### The Dental Expert Intervention and Opinion

Similarly, the expert also has the task of initially contextualizing and organizing the case in accordance with the evidence decision. Further the questions posed by the court need to be answered in detail, carefully, impartially, comprehensibly, conclusively, and without contradiction in the written expert opinion. 3 25

Consequently, a dental expert opinion is concerned with providing a professional answer to the questions posed by the judge from a dental perspective. The aim is for the judge or judges to have the dental expertise required to make a decision after studying the written expert opinion. 25 The legal assessment of the facts is of course the responsibility of the judge. In order to prepare their expert opinion, the expert must review the available documents, potentially request further records via the court. Depending on the individual case, the expert might conduct a clinical examination of the patient. 3 25 In the case of special questions that cannot be answered directly on the basis of expertise and/or clinical experience, the expert must derive a researchable scientific question from the topic of evidence. After reviewing and evaluating the literature, the expert must then reflect on the results of his research and adapt them to the individual case. With regard to the content, his assessment must take into account the level of care to be assessed, the time of treatment and the freedom of methods. 3 26

### Aims and Scopes of the Article

As the legal regulations in the individual countries differ, the example of Germany was chosen because Germany is a constitutional state in which the separation of powers (legislative, executive, and judiciary) is implemented in an exemplary manner and therefore serves as a model for many countries. In the area of medical malpractice, the subspecialty title “Fachanwalt für Medizinrecht” (specialist lawyer for medical law) was introduced 20 years ago, which demonstrates the high level of professionalism in medical malpractice law. In addition, there have been various initiatives in Germany in recent years to improve the quality of dental expert reports. 1 9 It therefore can be expected that the requirements for dental court opinions are particularly high.

The aim of the review was to reflect on general aspects of dealing with literature and evidence in the context of dental expert activities and to make recommendations that can then be adapted to the respective legal system.

## Methods

A literature search was conducted using the PubMed and Cochrane Library databases and Google Scholar on the question “What evidence base should dental experts use to prepare a court report?” The search terms included “expert opinion”, “dentistry”, “expert”, “medical liability”, “malpractice”, and “evidence”. German- and English-language publications published in the period from January 1, 2000, to December 31, 2023, were included. No scientific publication was found that fully addresses the research question raised. Based on this search, the most relevant topics were identified in a subjective manner and are now listed and discussed in this article.

## Results

In the identified scientific articles that partly discussed relevant topics on the research question, the reason given for the paucity of literature is that court decisions are strongly influenced by the laws of a particular country, the timing and the country's health care system. 12 It is unanimously reported that legal disputes in the field of dentistry have increased and that it is desirable to address the issue in the interests of both dentists and patients. 12 At the same time, dentists are said to have insufficient knowledge of the legal aspects of the dental profession and dental liability. This negligence is often associated with negative personal consequences for the dentists concerned. 2 27

The Association of the Scientific Medical Societies in Germany (AWMF) has published a guideline on medical expert witness assessment for Germany in 2019. 25 This guideline was written jointly by several medical societies in Germany. Among them, the German Society for Oral and Maxillofacial Surgery is the only dental specialist society that was involved. According to the objective of the guideline, emphasis was made that literature references, doctrinal opinions, and empirical knowledge are summarized by professionals experienced in writing expert opinions. 25 Even though the guideline was not created specifically for dentists, but rather for or other medical specialties, it provides helpful information for dental experts on the formal structure and drafting of an expert opinion and therefore was relevant for this on the issue.

There are only two German-language textbooks on dental expert witness assessment in the specified search interval. These is the textbook “ *The Dental Expert* ” by Oehler, 28 published in its second edition in 2003, and the book “ *Dental Treatment and Expert Opinion* ” by Münstermann, also published in its second edition and updated in 2009. 29 Oehler focuses on legal expert activities on behalf of the courts and presents a collection of dental court decisions from German civil courts. 28 Münstermann describes a special process of expert assessment that is conducted in some patients in need for dental prostheses with basic public health insurances in Germany. Even though these expert opinions are not written for the courts, the author provides important general information on the formal preparation of expert opinions. 29 At this point, the German reference book “ *Basics of Medical Assessment* ” by Becher and Ludolph should also be mentioned even though it does not address dental-specific topics. This book was designed in accordance with the curricular training of the German Medical Association “Basics of the medical expert opinion” and contains recommendations in some places that can be useful also for dental experts. 30

The literature search revealed some basic aspects for the formal setup of legal dental expert opinions and offers advice for the handling of the existing evidence.

## Fundamental Aspects

### The Dental Expert Opinion

A dental expert opinion must be formally distinguished from a sick certificate, from diagnostic and progress reports and from structured reports for other indications. 25 An expert opinion is written in a free form and is based on the available underlying evidence. Often it is based on a clinical examination of the patient by the expert. The special form of an expert report without a clinical examination is referred to as a file-based report. 25 A dental expert opinion is subject to formal requirements and numerous quality requirements. The medical expertise, the impartiality of the expert, and the general comprehensibility of the expert opinion must be emphasized. 3 25 Expert opinions should be neutral, impartial, objective, structured, thorough, bound by professional standards, independent, autonomous, transparent, comprehensible to laypersons, and delivered on time. 3 25

### The Dilemma between Clinical Findings and Absolute Medical Truth

In general, the evaluation of a dental treatment should be based on established and proven medical knowledge. If the answer to the questions of evidence requires the inclusion of medical hypotheses and the presentation of controversial views, this should be made clear in the written statement. 31 The expert is faced with the difficulty of having to consider subjective complaints presented by the patient as well as objective clinical and radiographic findings, such as two-dimensional X-rays and cone beam computed tomography. Often the usefulness and prognostic significance of these findings cannot be assessed using an absolute, generally binding standard. 31 Judges often demand the absolute medical truth. This question often cannot sufficiently be answered. For this reason, the questions should be answered with the best available scientific evidence. 31

### The Standard of Treatment Is Subject to Change

The dental specialist treatment standard is not regulated by law. The standard of care can be defined as obtainable quality standard derived from medical evidence and experience among a variety of accepted treatment options. 26 This standard continues to evolve. This change is due to new diagnostic methods, newly introduced surgical and treatment techniques, the publication of new research results, systematic reviews, meta-analyses, new materials, new technical devices and possibilities. In addition, there are new legal policies and procedures that affect practice. For this reason, the question of dental standards must always be assessed by the assessor from an “ex ante” perspective. It should be noted that today's standard of care can, in extreme cases, be tomorrow's treatment error. This means that the expert is required to go back in time to objectively assess the case. In particular, the literature referred to must have been published and known at the time of treatment. In this respect, the expert must refrain from citing the latest literature in his report. He is required to use the literature valid at the time of the treatment to be assessed as a basis and also to consider whether this was already common knowledge in the general dental practice. 3

### Challenges for Assessment of the Cases

For expert witness evaluation, a proper assessment appropriate to the dental intervention is required. 3 The reviewer must refrain from making legal statements. 25 This is the exclusive task of the legal profession. An assessment in the sense of “in dubio pro aegroto” is unacceptable. The preparation of an expert opinion is therefore by no means trivial, but remains a responsible, often difficult task. 31 The expert witness is bound to the nature and scope of their task and should only answer the questions that were specifically addressed. 25 In individual cases, the Code of Civil Procedure in Germany stipulates that the expert may request an explanation or amendment to the assignment before accepting the mandate. 25

## Sources of Evidence

### Transparency Contributes to the Overall Acceptance of the Expert Opinion

Literature references make the report more transparent for all parties involved, as the sources of professional information are disclosed, and thus technical contexts become comprehensible even for the so-called interested layperson. 3 It opens the possibility of conducting one's own research. In the case of everyday dental knowledge, which is directly available to every dentist, citations are considered superfluous. They would unnecessarily lengthen the expert opinion. Corresponding evidence in a few suitable places in the expert opinion can certainly contribute to the expert opinion being regarded as credible overall, as the sources are disclosed and the expert opinion is then more likely to be accepted by the parties. In these cases, a dental textbook or a widely used standard work could be used as a reference. Another important aspect is to keep the report comprehensible by translating technical dental terms in brackets when they are mentioned for the first time. However, a naïve simplification of complex dental contexts in the expert opinion should still be avoided.

### Role of National Peer-Reviewed Dental Journals

In order to better meet the interests of the court, it can be helpful to concentrate on specialist articles in the respective national language. This approach avoids additional linguistic hurdles in understanding the not always simple medical and scientific contexts and prevents misunderstandings. National peer-reviewed journals sometimes provide an easily understandable review of the current essence of international publications.

One disadvantage of national journals in languages that are not listed in international directories is the limited detectability in systematic evidence searches.

### Role of National Guidelines

In the context of quality assurance measures, national guidelines play an increasingly important role as evidence-based recommendations for action in dentistry. 32 This also applies to the preparation of dental expert witness assessments, which should be evidence based. Guidelines can therefore generally be helpful in connection with assessments but are of course no substitute for expert opinions. 33

In dentistry, the AWMF has produced several medical and dental guidelines over the past 10 years. 32 These guidelines are available open access. The hierarchical subdivision of guidelines in Germany is based on qualitative development stages (S1–S3). S1 guidelines are based on informal consensus among experts and are therefore not subject to a systematic evidence development process. S3 guidelines represent the highest level of development. They are characterized by a high level of evidence through systematic literature research and at the same time achieve a high degree of consensus among the representative panel of experts. 32 As of May 2023, 44 dental guidelines are available in Germany, 26 of which are rated at the highest level of development (S3). 32 This standard is filled in by dental facts, which are usually assessed by the dental expert witness. Guidelines as intraprofessional recommendations for diagnostic and therapeutic procedures for certain situations have increasingly become the subject of legal interest. 33 It must therefore be assumed, particularly in the case of guidelines with a high level of evidence, that nonconformity with the guideline recommendation can certainly have a negative impact in the evaluation of treatment errors for the dentist.

It should therefore be noted that the dental expert witness should be familiar with the national dental guidelines or should research whether corresponding guidelines that affect the disputed issue are available. The expert witness should then interpret these existing recommendations in the context of the individual case. If there are no national guidelines on the respective topic, it may of course also be necessary and helpful to incorporate international consensus papers from the relevant professional associations.

### Systematic Evidence Searches in Literature Databases

In a systematic literature search with PubMed and other scientific databases, there is a risk of misinterpretation of the literature due to considerations of standard patients in research (mean value, inclusion and exclusion criteria) and the individual case. Here, the expert witness is required to translate the results of his literature research—similar to the available guidelines—back into the context of the case and the knowledge of the dental practice at the time. The search function can be used in the same way as for a scientific question. For example, it makes sense to break down the question into subaspects, such as in the PICO scheme (Patient, Intervention, Comparison, and Outcome). 34 The search can begin with an aspect from which the fewest hits are to be expected. Depending on the number of hits, the search can be narrowed down using the “and”-function or expanded using the “or”-operator. The Medical Subject Headings terms matching the query can be identified among the search results. 34 Limiting the search by article type, language (English and corresponding national language) and publication date are recommended.

A summary of the categories of the written formal report mentioned earlier and the suggested sources of evidence (standard textbooks, national peer-reviewed dental journals, guidelines, and international peer-reviewed dental journals) are presented in Table 1 .

**Table 1:** Reason for citation with adequate sources of dental literature

	Standard textbooks	National peer-reviewed dental journals	Guidelines	International peer-reviewed dental journals
General explanation of the clinical management	++	+	+	−
Definition of medical standards	++	−	++	+
Medical malpractice	+	+	++	+
Gross medical malpractice	++	+	++	+
Scientific justification of causal relationships	−	−	+	++

Note: ++ denotes particularly appropriate, + appropriate, and - not or less recommended.

## Discussion

Based on a literature review, our article presents a number of aspects of the appropriate handling of evidence in dental expert opinions. During the research, it quickly became apparent that the handling of evidence in dental expert opinions represents a research desideratum. The results section can therefore only mention a few relevant aspects in form of a narrative review. Qualitative approaches such as expert interviews or focus group discussions with experienced experts or other relevant persons working in medical law could be a topic for future research to qualitatively evaluate and address the topic.

The primary task of the dental expert witness is to make the case legally applicable for the court through his professional expertise and the targeted answering of the provided questions of evidence. The expert therefore has a prominent position. With his expert opinion, he indirectly contributes to legal certainty in the field of dentistry, oral and maxillofacial medicine, as the judge follows the expert's statements and makes decisions on that basis in the majority of cases. 16

The general considerations on the available sources of written evidence have shown that standard dental textbooks and national and international dental journals are useful sources of knowledge. Furthermore, when the dental expert witness refers to a source, this can contribute to a better understanding and an increase in general acceptance of the report. Of course, the results of the literature search need to be brought into the context of accepted clinical dental practice and a certain adaptation of the findings derived from the published literature to the individual case must take place. In this context, it is easy to understand that dental guidelines and standard textbooks can never replace an expert opinion. 35

The reason is that evidence cannot replace individual clinical expertise and the interpretation of data. If one were to rely exclusively on published results from studies when making clinical decisions, this would imply that “cookbook medicine” is practiced. 36 In addition, the expert opinion is subject to the special feature that the expert was not involved during the treatment in question. In order to be able to perform the aforementioned interpretation work properly, the training of legal dental assessments and the collegial exchange among dental legal experts remains crucial. 24 37 The literature search forms the basis for the subsequent assessment of the case by the legal dental expert. In principle, the expert literature search does not initially differ from the procedure for purely scientific questions. The only restriction is that the literature used must already have been published at the time of the disputed treatment, 3 and the published knowledge must also already be considered the general standard of care at the time. On the other hand, it could be argued that a corresponding reference can be found for almost any “expert opinion,” no matter how far-fetched.

As assessment is not a part of teaching at the universities during dental studies, the knowledge should be acquired at a later stage of professional practice in institutions that offer continuing medical education courses. Studies on real example cases are beneficial. 9 37 In this context, it should not go unmentioned that in Germany both scientific dental societies and professional dental associations have been offering corresponding training courses for a few years or are currently planning them.

In this context, the introductory article on dental liability by Blau and Levin, which looks at tort law and the interpretation of negligence in different countries, needs to be mentioned. 23 The article gives an overview on the main different liability regimes and the legal elements that need to be proven in each regime to obtain compensation for negligence in the field of dentistry. In times of rising medical malpractice lawsuits, the authors argue that it is crucial that dentists are aware of the basic legal concepts of medical malpractice. 23 It is also beneficial for dental associations to publish national recommendations for legal expert opinions, such as the Australian Dental Association did for Australia. 38 The document, which is only five pages long, regulates some formal matters regarding the expert examination of the patient and provides a number of general instructions on how the written expert opinion should be drafted. Ultimately, it remains unclear whether dentists providing expert opinions adhere to these guidelines. As this document is freely available on the Internet for anyone to access, it can be assumed that such statements are also viewed by judges and lawyers. In this respect, it can be assumed that in the event of deviations from the procedure described there, lawyers, as a group of people oriented toward authoritative documents, will very likely draw attention to differences.

## Conclusion

Based on a literature search, our narrative review presents several important aspects of dealing with evidence in the preparation of dental court reports. It is advisable for the dental expert to consider the national scientific statements and guidelines of the scientific dental societies when preparing his or her expert opinion. Further, standard dental textbooks and scientific studies can also be helpful when preparing an expert opinion.
